# *Caenorhabditis Elegans* and Probiotics Interactions from a Prolongevity Perspective

**DOI:** 10.3390/ijms20205020

**Published:** 2019-10-10

**Authors:** Marianna Roselli, Emily Schifano, Barbara Guantario, Paola Zinno, Daniela Uccelletti, Chiara Devirgiliis

**Affiliations:** 1 Research Centre for Food and Nutrition, CREA (Council for Agricultural Research and Economics), 00178 Rome, Italy; marianna.roselli@crea.gov.it (M.R.); barbara.guantario@crea.gov.it (B.G.); paola.zinno@crea.gov.it (P.Z.); 2 Department of Biology and Biotechnology “C. Darwin”, Sapienza University of Rome, 00185 Rome, Italy; emily.schifano@uniroma1.it

**Keywords:** ageing, nematode, immunosenescence, oxidative stress, lifespan, probiotic bacteria, pathogen protection

## Abstract

Probiotics exert beneficial effects on host health through different mechanisms of action, such as production of antimicrobial substances, competition with pathogens, enhancement of host mucosal barrier integrity and immunomodulation. In the context of ageing, which is characterized by several physiological alterations leading to a low grade inflammatory status called inflammageing, evidences suggest a potential prolongevity role of probiotics. Unraveling the mechanisms underlying anti-ageing effects requires the use of simple model systems. To this respect, the nematode *Caenorhabditis elegans* represents a suitable model organism for the study of both host-microbe interactions and for ageing studies, because of conserved signaling pathways and host defense mechanisms involved in the regulation of its lifespan. Therefore, this review analyses the impact of probiotics on *C. elegans* age-related parameters, with particular emphasis on oxidative stress, immunity, inflammation and protection from pathogen infections. The picture emerging from our analysis highlights that several probiotic strains are able to exert anti-ageing effects in nematodes by acting on common molecular pathways, such as insulin/insulin-like growth factor-1 (IIS) and p38 mitogen-activated protein kinase (p38 MAPK). In this perspective, *C. elegans* appears to be advantageous for shedding light on key mechanisms involved in host prolongevity in response to probiotics supplementation.

## 1. *Caenorhabditis Elegans* as a Model System to Study Prolongevity

**Figure 1:**
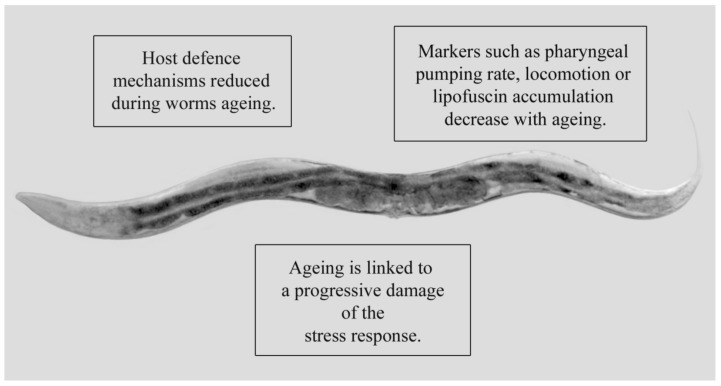
Micrograph representing the model organism *Caenorhabditis elegans* (the head is on the left; the tail is on the right). The principal biomarkers and physiological traits associated with ageing are described in the squares. Magnification: 5×.

*Caenorhabditis elegans* is a small nematode widely used as a model system for different biological studies because of its many advantages. It is characterized by transparency, a short life cycle, ease of cultivation, and availability of large sets of mutants [1]. Worms can be grown cheaply and in large numbers on agar plates and they are normally fed bacteria, although they can be also fed yeasts. In addition, even if *C. elegans* is considered a simple organism, many of the molecular cascades controlling its development are also found in more complex organisms, like humans [2,3]. Nematode lifespan is a parameter that can be influenced by genetic and environmental factors, including nutritional stimuli. The genes involved in lifetime regulation are associated with different molecular pathways, evolutionarily conserved, that modulate ageing processes [4], such as insulin/insulin-like growth factor-1 (IIS) [5] and p38 mitogen-activated protein kinase (p38 MAPK) pathways [6]. For these reasons *C. elegans* represents a suitable model organism for ageing studies and for evaluating the impact of nutritional stimuli on prolongevity. Indeed, different bacterial feedings can play an important role in the regulation of nematode lifespan by inducing specific host responses [7]. In particular, while some pathogens shorten worm viability, several probiotic strains show beneficial effects, prolonging lifespan and leading to a delay in ageing [8,9]. It has been widely reported that these effects correlate to the host defense responses and stress resistance of *C. elegans* [10]. Indeed, ageing is characterized by progressive damage of the stress response and cellular machine. In nematodes different ageing biomarkers could be studied to evaluate the effects of a diet. Indeed, pharyngeal pumping rate, locomotion ability, body size and intestinal lipofuscin autofluorescent granules are the most examined markers, thanks to the ease of analysis [11,12] (Figure 1). *C. elegans* is therefore considered as one of the best model systems used to study longevity and to screen bacteria showing probiotic properties and anti-ageing effects [13].

## 2. Probiotics: Characteristics and Relevance to Ageing

Probiotics are commonly defined as “live microorganisms that, when administered in adequate amounts, confer a health benefit to the host” [14]. The majority of species known to have probiotic properties belong to the genera *Lactobacillus* and *Bifidobacterium*, commonly found in the gastrointestinal tract of humans and animals and thus generally regarded as safe. However, also members of other bacterial genera can have documented health benefits, such as *Bacillus*, *Enterococcus* as well as the yeast *Saccharomyces*. It is widely recognized that the health benefits of probiotics are strictly strain-specific, consequently distinct strains belonging to the same species can have different effects. For this reason, accurate characterization of novel potentially probiotic strains is very important. Amongst various possible mechanisms of action, probiotics are believed to exert their effects by production of antimicrobial substances, competition with pathogens for adhesion sites and nutrients, enhancement of host mucosal barrier integrity and immune modulation [15,16]. Thus, the beneficial activities of probiotics are attributable to three main core benefits: supporting a healthy gut microbiota, a healthy digestive tract and a healthy immune system [17].

In the context of ageing, several physiological changes affecting the immune and digestive systems as well as gut microbiota composition lead to a physiological low-grade inflammatory status called, “inflammageing” [18,19], which can be potentially counteracted by probiotic interventions [20]. Indeed, perturbations of gut microbiota composition and immune function associated with ageing can favor the growth of pathogens and increase the susceptibility to gut-related diseases [21], affecting members of the health-promoting bacteria resident in the gut. In particular, a reduction of numbers and species of bifidobacteria has been reported in older persons [22,23]. At this stage of life, probiotics exert several beneficial effects for the host by protecting against pathogenic bacteria and viruses, enhancing immune function, counteracting intestinal inflammatory diseases, and improving metabolic functions and the lipid profile [24,25]. 

One of the major issues related to the study of probiotics is the need of appropriate, simplified in vivo models representing useful and less expensive screening tools to identify probiotic strains from a large number of microbial candidates. To this respect, the nematode *Caenorhabditis elegans* is becoming an increasingly valuable in vivo model to study host-probiotic interactions to enhance lifespan [26].

## 3. Review Methodology

We conducted a literature search on PubMed by using the following keywords: (1) *Caenorhabditis elegans* and probiotics; (2) *Caenorhabditis elegans* and lactobacilli; (3) *Caenorhabditis elegans* and bifidobacteria; and (4) *Caenorhabditis elegans* and lactic acid bacteria. Publication dates were restricted to the last ten years. The first search retrieved 46 results. The other searches produced a majority of overlapping results with the first one and some additional results, in particular: the second search retrieved six more results; the third one two more results and the fourth one three more results. Following this initial search, eight articles were excluded, on the basis of their low adherence to the description of probiotic activities in *C. elegans*, which was the main focus of our search. Thus, finally 49 articles were carefully evaluated for the review preparation. Table 1 shows a list of the microorganism strains described in the selected literature to exert a probiotic activity in *C. elegans*. The majority of the tested species belonged to the *Lactobacillus* genus, with 16 different species and 35 strains, followed by the *Bifidobacterium* genus, with 4 species and 6 strains. Then we found 2 *Pediococcus*, 2 *Weissella* and 2 *Enterococcus* species, and finally *Bacillus, Butyricicoccus, Megasphaera, Clostridium, Propionibacterium, Escherichia* and *Kluyveromyces*, with one species each. Of note, only *Kluyveromyces marxianus* belongs to the Ascomycota phylum within the yeast kingdom.

Among the collected papers, those taking into account the effect of probiotic supplementation on nematode lifespan were further selected and analyzed to evaluate important parameters related to ageing, such as oxidative stress, immune system and susceptibility to pathogen infection, which are known to be involved in immunosenescence. This condition refers to the gradual deterioration of the immune system brought on by age progression. It involves both the host capacity to respond to infections and the development of long-term immunological memory. Immunosenescence can be considered as a crucial contributory factor to the increased occurrence of morbidity and mortality among the elderly. This age-related immune deficiency is ubiquitous and found in both long- and short-living species, and it is characterized by a particular “remodeling” of the immune system, induced by oxidative stress [27]. Together with inflammageing, immunosenescence is suggested to stand at the origin of the majority of elderly-related alterations, such as infections, cancer, autoimmune disorders, and chronic inflammatory diseases [28]. The present review focuses on the role of probiotics on *C. elegans* age-related parameters, with particular emphasis on oxidative stress, immunity and inflammation, and protection from pathogen infections.

## 4. Mechanisms Involved in *C. elegans* Lifespan Extension Induced by Probiotics

### 4.1. Description of the Main Pathways

The principal pathways involved in lifespan control, oxidative stress, regulation of immune response and defense against pathogen infection in *C. elegans* include the IIS pathway and p38 MAPK pathway [71]. Each pathway is composed of a cascade of signaling molecules that finally activate/regulate the transcription of specific target genes. In particular, the IIS pathway is initiated by the activation of dauer formation (DAF)-2, an insulin/insulin-like growth factor-1 receptor ortholog, subsequently triggering a cascade of phosphorylation events that activate specific kinases and downstream mediators. These include phosphatidylinositol 3-kinase AGE-1, phosphoinositide-dependent kinase (PDK)-1, and various serine/threonine protein kinases (AKT-1, AKT-2, and SGK-1), culminating in phosphorylation of DAF-16, a protein belonging to class O of the forkhead transcription factors (FOXO), resulting in its inactivation [72]. On the contrary, in the presence of heat stress, anoxia, oxidative stress, starvation, and infections, the IIS pathway is down-regulated and DAF-16 migrates to the nucleus, where it switches on the expression of specific target genes, that contribute to several cellular processes, from apoptosis to stress resistance, prolongevity and anti-ageing [58,73]. The nuclear translocation of DAF-16 leads to both up-regulation and down-regulation of large sets of genes, referred to as class I and II, respectively [5]. The IIS signaling pathway transcriptionally regulates many genes involved also in the immune responses, closely linked to longevity in *C. elegans*.

The p38 MAPK pathway is the most ancient signal transduction cascade in nematode immunity and plays a central role in *C. elegans* response against different pathogens, as it does in mammals. The p38 MAPK pathway is required for the activation of a set of immune effectors necessary to maintain a basal level of immune function and it is also involved in lifespan extension [67,74]. A neuronal symmetry (NSY)-1–SAPK/ERK kinase (SEK-1)–p38 mitogen-activated protein kinase ortholog (PMK)-1 p38 MAPK cascade (MAPKKK-MAPKK-MAPK, respectively) was elegantly identified as a key component of the *C. elegans* immune response [6].

Such signaling pathways are evolutionarily conserved in different animal species, from nematodes and flies to higher vertebrates and mammals. Evidence suggests that these pathways are relevant also to mammalian aging [75]. In particular, human studies conducted on centenarians highlighted an important role of the IIS pathway in setting lifespan, since associations have been found between polymorphisms in IIS genes and longevity [76].

**Figure 2:**
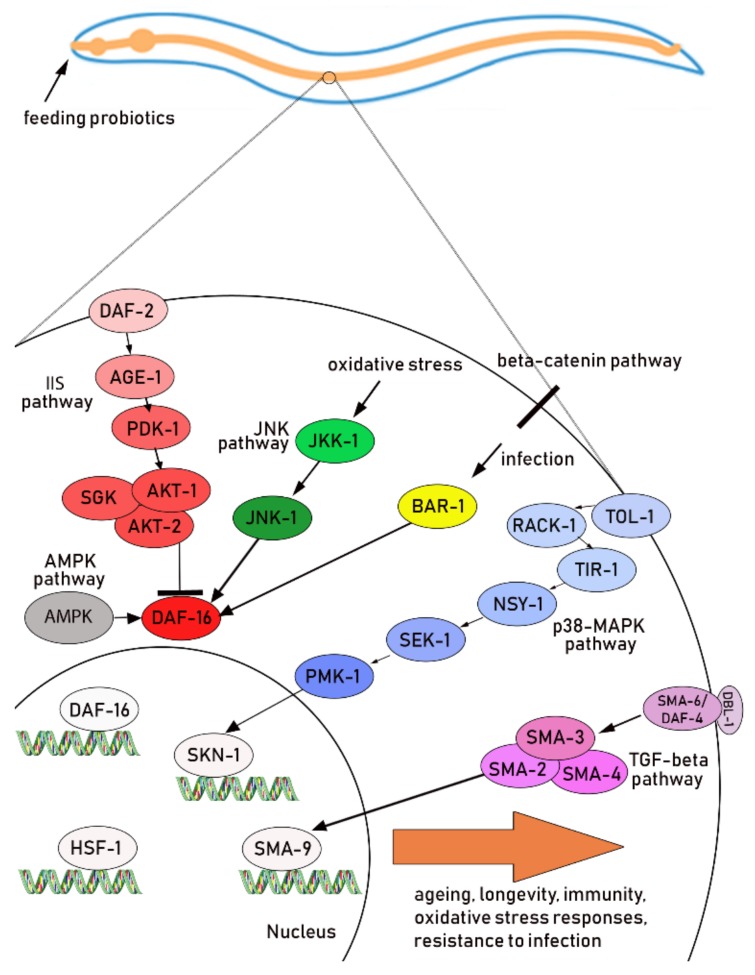
Schematic representation of the most common signaling pathways influenced by probiotic strains employed in *C. elegans* studies. Each pathway is represented by a distinct color gradient. The list of single bacterial strains influencing the different pathways is reported in Table 1. Abbreviations used: AMPK: 5′ AMP-activated protein kinase; AKT-1/2: serine/threonine protein kinase orthologs; BAR-1: beta-catenin/armadillo Related-1; DAF: dauer formation; DBL-1: DPP/BMP-Like-1; HSF-1: heat-shock transcription factor-1; IIS: insulin/insulin-like growth factor-1; JKK-1: c-Jun N-terminal kinase kinase; JNK-1: c-Jun N-terminal kinase; MAPK: mitogen-activated protein kinase; NSY-1: neuronal symmetry-1; PDK-1: phosphoinositide-dependent kinase 1; PMK-1: p38 mitogen-activated protein kinase-1 ortholog; RACK-1: receptor activated protein C kinase; SEK-1: SAPK/ERK kinase-1; SGK-1: serine/threonine protein kinase ortholog; SKN-1: skinhead family member-1; SMA: small; TIR-1: toll interleukin-1 receptor-1.

Probiotic strains used in *C. elegans* studies have been shown to act through one or more of the above mentioned signaling pathways (Figure 2 and Table 1).

### 4.2. Oxidative Stress Response

Oxidative stress plays a detrimental role in different organisms. Normally, antioxidant defenses protect cells by removing reactive oxygen species (ROS). During ageing, on the other hand, ROS and other products of oxygen metabolism accumulate damaging proteins, lipids and DNA, and weaken antioxidant defenses [77]. In *C. elegans* animal model, several studies have been carried out to understand the mechanisms through which probiotics can influence ageing, by activating different longevity signaling pathways related to oxidative stress resistance. Prolongevity and oxidative stress responses in *C. elegans* fed probiotics are induced via mechanism(s) that can be DAF-2/DAF-16-dependent or a result of a cross talk among different pathways.

Among the tested strains, *Bifidobacterium longum* strain BR-108 has been shown to increase worm lifespan following H_2_O_2_-induced oxidative stress, through activation of IIS pathway. After the cascade activation, DAF-16 seems to co-localize with the heat-shock transcription factor (HSF)-1 in the nucleus, inducing the transcription of *hsp-16.2* and *hsp-70* that are involved in stress responses and longevity [59].

Similarly, *Lactobacillus rhamnosus* CNCM I-3690 and *Bifidobacterium animalis* subsp. *lactis* CECT8145 strains stimulated a strong resistance to oxidative stress in *C. elegans*, which was in part dependent on the IIS pathway [49,53]. On the other hand, *Lactobacillus gasseri* SBT2055 (LG2055) has been reported to promote a prolongevity effect in *daf-2* and *daf-16* mutant worms [9], thus demonstrating that prolongevity and enhancement of stress resistance were DAF-16-independent. These phenotypes occur rather by triggering the p38 MAPK pathway, which culminates with the nuclear translocation of the transcriptional factor skinhead family member (SKN)-1 [9]. SKN-1, an ortholog of the mammalian Nrf2, induces the expression of target genes involved in oxidative stress resistance and it is responsible for the beneficial effect exerted by several other probiotic microbes [37,62,69].

*Bifidobacterium longum subsp. infantis* (formerly *B. infantis*) strain ATCC15697 resulted to extend wild-type nematode lifespan, but it failed to prolong the lifespan of *pmk-1, skn-1* and also *daf-2* mutants, demonstrating the involvement of p38 MAPK and IIS signaling pathways, both modulating SKN-1 activation [56,78].

It has been reported that the c-Jun N-terminal kinase (JNK) family, a subgroup of the MAPK superfamily, phosphorylates DAF-16 at a different site with respect to DAF-2-mediated phosphorylation, resulting in its nuclear translocation [79]. 

Similarly, AAK-2, which is one of the two alpha-catalytic subunits of 5′-AMP-activated protein kinase (AMPK), can directly phosphorylate DAF-16, triggering prolongevity and oxidative stress responses. Analysis of lifespan and gene expression of worms fed *Weissella koreensis* or *W. cibaria*, demonstrates that some *Weissella* species promote longevity in *C. elegans* by inducing oxidative stress responses through activation of DAF-16 via the JNK and AMPK pathways [68].

Several detoxifying enzymes are induced by different transcription factors in response to oxidative stress. Two of these are the superoxide dismutase (SOD) and the glutathione S-transferase (GST), which detoxify ROS [80]. Moreover, oxidative stress causes the activation of the transcriptional factor HSF-1, which also regulates lifespan, and activation of JNK pathway. Zhao and coworkers demonstrated that *Bifidobacterium longum* BB68, isolated from a centenarian subject, was able to increase lifespan and oxidative responses in *C. elegans,* through increased expression of *sod-3* gene, mediated by the toll interleukin-1 receptor (TIR)–JNK signal transduction pathway resulting in DAF-16 nuclear translocation [58]. Specifically, this highly conserved pathway consists of a TIR-domain protein, TIR-1, activating JNK-1 through phosphorylation. In turn, JNK-1 phosphorylates DAF-16, which migrates to the nucleus.

As stated above, intracellular ROS represent an important marker to analyze the extent of oxidative stress, and some probiotic strains have been shown to reduce their level in *C. elegans*. *L. fermentum* MBC2, in addition to lifespan extension and anti-ageing effects, induced a reduction of ROS levels and an increased expression of detoxifying enzymes, such as GST-4, paralleled by an amelioration of the other ageing biomarkers, such as locomotion activity, pumping rate and lipofuscin granules [36].

### 4.3. Immune Response and Pathogen Protection

Several candidate probiotic bacteria analyzed in this review have also been demonstrated to affect immunity and inflammation pathways in *C. elegans*. Immune response and lifespan are tightly linked in *C. elegans*. Among different molecular pathways shared with higher organisms, innate immunity of *C. elegans* shows many aspects similar to humans. Although the nematode does not have a cell-mediated immune system, it possesses innate immune defense mechanisms that are evolutionarily conserved [74]. In particular, *C. elegans* possesses different pathways associated with immunity, including the above mentioned p38 MAPK and IIS pathways, but also the trasforming growth factor-beta (TGF-beta) [52,71,74,81] and the beta-catenin signaling pathways [82], which can be induced by probiotics (Figure 2).

Immune responses to bacteria are mediated by interaction of specific microbial cell wall structures, (microbial associated molecular patterns, MAMPs), such as peptidoglycan, teichoic acids and lipopolysaccharides, with host receptors, in particular toll-like receptors (TLRs). In mammals different TLRs have selective specificity for the different MAMPs, while in *C. elegans* a unique TLRs homolog, TOL-1, has been identified so far [83]. The interaction of a TLR with its microbial ligand activates several signaling pathways, including p38 MAPK, resulting in the transcription of genes necessary to mount the defense mechanism in the host. The main cell wall MAMPs share a common basic structure among different bacterial species, both pathogens and probiotics, but various subtle chemical modifications present in the different species or strains can contribute to the strain-specific properties of probiotics. This also implies that the final outcome of the TLR activation depends on the type of interacting microorganism, meaning that a MAMP from one bacterial species can activate a certain TLR, while a similar MAMP from another species, or strain, can down-regulate the same TLR signaling [84]. The involvement of nematode TOL-1 in the regulation of prolongevity effect exerted by *Bifidobacterium longum* subsp. *infantis* (formerly *B. infantis*) strain ATCC15697 was recently demonstrated [57].

The majority of probiotic strains were employed in *C. elegans* to verify their prolongevity effects in the context of protection from pathogen infection, through killing assays. Some others were tested on lifespan extension in normal conditions. The use of nematode functional mutants or the analysis of gene expression profile by RT-qPCR/microarray allowed the elucidation of the molecular players acting as targets of probiotic action. Many human pathogens, such as *Pseudomonas aeruginosa*, *Salmonella enterica*, *Staphylococcus aureus*, *Klebsiella pneumoniae*, enterotoxigenic *Escherichia coli, Yersinia enterocolitica* and *Listeria monocytogenes*, can cause nematode death. It is known that pathogen infection induces worm innate immune responses, consisting in the production of several antimicrobial proteins, whose expressions are regulated by signaling pathways involved in the defense against harmful bacteria [74]. Such antimicrobial proteins include lysozyme (LYS) family, and C-type lectins (CLEC). As mentioned above, *C. elegans* lacks a cell-mediated immune system and the production of antimicrobial peptides is, therefore, the outcome of its innate immunity to counteract infections [85]. As an example, *Bacillus subtilis* NCIB3610, which forms a biofilm contributing to nematode prolongevity, specifically stimulated *lys-2* expression, increasing *C. elegans* resistance to *P. aeruginosa* infection [86].

Zhou and coworkers reported that *L. reuteri* CL9 induced the expression of antimicrobial peptide genes *clec-60* and *clec-85*, involved in the protection of nematodes against enterotoxigenic *Escherichia coli* (ETEC) infection [51]. Similarly, *L. zeae* LB1 induced the production of antimicrobial peptides and defensive molecules, such as LYS-7 and CLEC-85, through the p38 MAPK and IIS pathways, enhancing resistance of *C. elegans* to ETEC infection [52]. The p38 MAPK pathway was also activated by *L. acidophilus* NCFM, employed for protecting nematodes against the Gram-positive pathogens *Staphylococcus aureus* and *Enterococcus faecalis* [29], as well as by *L. casei* LAB9, which displayed protection against *Klebsiella pneumoniae* infection. In particular *L. casei* LAB9 activates TLR and triggers the PMK-1/p38 MAPK pathway through the up-regulation of receptor activated protein C kinase (RACK)-1, an adaptor molecule that plays a critical role in the host defence and survival [32].

The p38 MAPK pathway is also involved in protection against *Legionella pneumophila* infection promoted by *Bifidobacterium longum* subsp. *infantis* (formerly *B. infantis*) ATCC 15697 via PMK-1 [55], as well as in stimulation of *C. elegans* host defense by six foodborne strains of *Bacillus licheniformis* [87]. Moreover, Kwon et al. (2016) described that *Propionibacterium freudenreichii* KCTC 1063, isolated from a dairy product, increased resistance against *Salmonella* typhimurium, through the activation of SKN-1, upon phosphorylation by PMK-1 [67].

*L. acidophilus* NCFM immune stimulation involved also the beta-catenin pathway through the beta-catenin/armadillo related (BAR)-1 mediator, indicating that different signaling pathways can act in parallel to promote immunity [29].

On the other hand, the IIS signaling pathway was influenced by *Clostridium butyricum* MIYAIRI 588 (CBM 588), which was able to confer resistance to *S. aureus* and *S. enteric* infection through DAF-16-dependent class II genes [62]. Two other genes implicated in the defense response and the innate immune response through the IIS pathway, *acdh-1* and *cnc-2*, were up-regulated by heat-killed *L. plantarum* LP133 and *L. fermentum* LF21, protecting worms against Gram-negative pathogens *Salmonella* typhimurium and *Yersinia enterocolitica* [39].

The evidence that the protective activity of different *Lactobacillus* species can be directed either to Gram-positive or Gram-negative bacteria indicates that probiotic effects are species- and strain-specific, as explained above, concerning TLR-MAMP interactions. To this respect, the above-mentioned *Lactobacillus acidophilus* strain NCFM, while active against Gram-positive bacteria, displayed a minimal inhibitory effect on Gram-negative infection with *P. aeruginosa* or *S. enterica* [29].

In the absence of pathogen infection, lifespan extension exerted by *L. salivarius* DSM 20555 resulted to be dependent on the up-regulation of *lys-7* and *thn-2* genes, encoding LYS and an immune effector member of the thaumatin family, respectively, in a DAF-16-independent manner, suggesting the involvement of pathways other than IIS signaling [47]. In line with this evidence, *Butyricicoccus pullicaecorum* KCTC 15070 and *Megasphaera elsdenii* KCTC 5187 prolonged *C. elegans* lifespan by activating nuclear receptor signaling and the innate immune system in a TGF-beta pathway-dependent, but IIS pathway-independent manner. The signaling involves DPP/BMP-Like (DBL)-1, a mediator of the TGF-beta pathway, which binds to TGF-beta receptors, such as SMA-6/DAF-4, on the cell membrane and activates transcription of specific target genes related to antibacterial defense, inducing the production of antimicrobial peptides, such as CLEC, LYS and lipase [61].

## 5. Conclusions

*C. elegans* represents a valuable in vivo model for studying how probiotics interact with the host and what mechanisms are involved in prolongevity. Its numerous tools and the possibility of genetic approaches has allowed advances in understanding these interactions. *C. elegans* shows highly conserved pathways through which the host responds to microbes, revealing cross-talk regulating longevity, ageing, stress resistance, and innate immune responses. The unique opportunity to manipulate its diet renders *C. elegans* a powerful model organism for understanding the effect of bacteria on these interconnected processes. Moreover, it also represents a low expensive screening tool to identify novel probiotic strains from a large number of microbial candidates. The picture emerging from this review evidences that several probiotic strains are able to exert anti-ageing effects in nematodes, by acting on common, conserved molecular pathways, such as IIS and p38 MAPK. The cell wall components of probiotic bacteria are thought to be primarily responsible for immunostimulation, as clearly demonstrated for TLRs-MAMPs interactions. Moreover, key ageing- and stress-associated regulatory elements, such as DAF-16/FOXO and HSF-1 transcription factors, also emerged as common targets of probiotic activities. However, other important mediators still need to be identified and characterized. In this perspective, *C. elegans* therefore appears to be advantageous to unravel key mechanisms involved in host prolongevity in response to probiotics supplementation. Since these mechanisms appear to be conserved across several species, the possibility of promoting the longevity of humans through the consumption of probiotics is gaining increasing attention. Indeed, probiotic supplementation has been suggested to slow or reverse the age-related changes in intestinal microbiota composition, as well as the gradual decline of immune function in elderly, thus lowering the risk of associated age-related morbidities. Nevertheless, future studies are needed to deepen insight on the effect of probiotics on longevity in mammals.

## Figures and Tables

**Table 1:** List of microbial strains reported in the selected literature to exert a probiotic activity in *C. elegans*.

Genus	Species	Strain(s)	Nematode Signaling Pathway(s) Influenced	References
*Lactobacillus*	*acidophilus*	NCFM	p38 MAPK beta-catenin	[29]
	*brevis*	SDL1411	unknown	[30]
	*casei*	CL11 LAB9	unknown p38 MAPK	[31] [32,33]
	*coryniformis*	H307.6	unknown	[34]
	*delbrueckii*	*bulgaricus* ATCC11842; *lactis* LMG6401; *lactis* 23	unknown	[35]
	*fermentum*	MBC2 JDFM216 LA12 LF21	unknown p38 MAPK unknown IIS	[36] [37] [38] [39]
	*gasseri*	SBT2055	p38 MAPK	[9]
	*helveticus*	NBRC15019	unknown	[40]
	*murinus*	CR147	unknown	[41]
	*paracasei*	28.4	unknown	[42]
	*plantarum*	CAU1054; CAU1055; CAU1064; CAU1106 JDFM60; JDFM440; JDFM970; JDFM1000 CJLP133 K90 NBRC15891	unknown unknown IIS unknown unknown	[43] [44] [38,39] [45] [40]
	*pentosus*	D303.36	unknown	[34]
	*reuteri*	CL9 S64 DSM 20016	unknown unknown unknown	[46] [31] [47]
	*rhamnosus*	R4 CNCM I-3690 NBRC14710	unknown IIS unknown	[48] [49] [40]
	*salivarius*	FDB89 DSM 20555	unknown unknown	[50] [47]
	*zeae*	LB1	p38 MAPK IIS	[51,52]
*Bifidobacterium*	*animalis subsp. lactis*	CECT8145	IIS	[53]
	*breve*	UCC2003	unknown	[54]
	*Infantis* ^1^	ATCC15697	p38 MAPK IIS	[40,55,56,57]
	*longum*	ATCC15707 BB68 BR-108	unknown JNK IIS	[29,40] [58] [59]
*Bacillus*	*licheniformis*	141	unknown	[60]
*Butyricicoccus*	*pullicaecorum*	KCTC 15070	TGF-beta	[61]
*Clostridium*	*butyricum*	MIYAIRI 588 (CBM 588)	IIS	[62]
*Enterococcus*	*faecalis*	MMH594 Symbioflor^®^	p38 MAPK beta-catenin unknown	[63] [64]
	*faecium*	L11 E007	TGF-beta p38 MAPK p38 MAPK beta-catenin	[65] [63]
*Escherichia*	*coli*	Nissle 1917	unknown	[66]
*Megasphaera*	*elsdenii*	KCTC 5187	TGF-beta	[61]
*Pediococcus*	*acidilactici*	DSM 20284 DM-9	unknown unknown	[47] [30]
	*pentosaceus*	SDL1409	unknown	[30]
*Propionibacterium*	*freudenreichii*	KCTC 1063	p38 MAPK	[67]
*Weissella*	*cibaria*	KACC11845	JNK AMPK	[68]
	*koreensis*	KACC11853	JNK AMPK	
*Kluyveromyces*	*marxianus*	CIDCA 8154	p38 MAPK	[69]

^1^ The current adscription is *Bifidobacterium longum* subsp. *infantis* [70].

## Abbreviations

AMPK	5′-AMP-activated protein kinase
CLEC	C-type lectins
DAF	Dauer Formation
ETEC	Enterotoxigenic *E. coli*
FOXO	Forkhead box O
GST	Glutathione S-transferase
HSF-1	Heat-Shock transcription Factor-1
IIS	Insulin/insulin-like growth factor-1
JNK	c-Jun N-terminal kinase
LYS	Lysozyme
MAMP	Microbial associated molecular pattern
p38 MAPK	p38 mitogen-activated protein kinase
PMK-1	Mitogen-activated protein kinase-1
ROS	Reactive oxygen species;
SKN-1	Skinhead family member-1
SOD	Superoxide dismutase
TGF-beta	Transforming growth factor-beta
TIR-1	Toll interleukin-1 receptor-1
TLR	Toll-like receptor
